# Working mothers during COVID-19: a cross-sectional study on mental health status and associations with the receipt of employment benefits

**DOI:** 10.1186/s12889-021-12468-z

**Published:** 2022-03-04

**Authors:** Melissa A. Kirwin, Anna K. Ettinger

**Affiliations:** 1 Milken Institute School of Public Health, The George Washington University, 950 NH Avenue NW, Washington, DC 20052 USA; 2 Department of Psychology, University of Pittsburgh, 210 South Bouquet Street, Pittsburgh, PA 15213 USA

**Keywords:** COVID-19, Mental health, Working mothers, Employment benefits, Family friendly benefits, Kessler 6 Psychological Distress Scale (K6), Short Warwick Edinburgh Mental Well-being Scale (SWEMWBS)

## Abstract

**Background:**

Beyond the sweeping physiological effects of COVID-19 infections in 2020 and 2021, the psychosocial impacts of lockdowns, social distancing, and the associated disruptions to daily life have brought on a simultaneous mental health crisis, particularly among many working mothers who are disproportionately balancing childcare, virtual schooling, and employment vulnerability. The aim of this study was to measure the mental health status of working mothers in the United States and associations with the provision of family-friendly employment benefits one year into the pandemic.

**Methods:**

Cross-sectional data were collected from a sample of working mothers in the U.S. using an online survey of mental health status and the receipt of employer-provided family-friendly benefits. Mental health was measured with the Kessler 6 (K-6) and the Short Warwick-Edinburgh Mental Wellbeing Scale (SWEMWBS). Perceived helpfulness of benefits was assessed through self-reported Likert-scale scores of 0 (not at all helpful) to 4 (extremely helpful) to determine mean helpfulness scores for benefit types. Multivariable logistic regression analyses were conducted to determine associations between receipt of employment benefits and serious mental illness (SMI).

**Results:**

A total of 728 participants met the study criteria, 83.7% were non-Hispanic/Latino white and 61.1% were 35–44 years of age. Among study participants, 54.3% (*n* = 395) and 21.8% (*n* = 159) reported psychological distress levels associated with moderate mental illness (MMI) and serious mental illness (SMI), respectively. Not receiving benefits was associated with a 50% increase in odds of SMI (*aOR* = 1.50, 95% *CI* [1.03–2.20], *p* = 0.036). Benefits perceived to be the most helpful for participants were flexible hours/schedule (3.5; *SD* ± 0.9), flexible work location (3.3; *SD* ± 1.1), and supplemental paid time off (3.1; *SD* ± 1.1), with mean scores above very helpful.

**Conclusion:**

Results suggest employment benefits may help support the mental health of working mothers and provide a call to action to employers and policy stakeholders to develop solutions addressing gaps in workplace benefits and mental health support for working parents, with sustainable reform in mind to mitigate employment benefit inequities exposed by the pandemic.

## Background

The broad physiologic, psychosocial, and economic impacts of the COVID-19 public health emergency have raised significant concerns for the mental health status of adults globally [1–3]. A critical subpopulation of concern is the 21.7 million working mothers that comprise 13% of the American labor force [4]. The strain on mothers of children school-aged and younger during the COVID-19 pandemic has spurred anxiety, depression, and the exacerbation of other mental health disorders [5–7]. Working mothers have faced unique challenges in simultaneously juggling employment and increased domestic responsibilities during the absence of stable childcare and schooling options [8]. Mothers’ stressors range from concerns over their children’s well-being to potential financial challenges related to wide-spread employment instability during the pandemic [9–11]. Approximately 1.9 million women have exited the labor force during the pandemic with peaks occurring in March and September 2020, coinciding with the transitions of children to home-based learning [12, 13].

COVID-19 pandemic-related job insecurity has been linked to an increased risk for poor mental health [14]. Additionally, for those individuals who lost or left a job during the pandemic and did not receive unemployment benefits, associations have been found with more unmet health-related social needs, such as food and housing, and poor mental health [15]. To partially alleviate the burden on Americans, including working mothers and their family members, the U.S. government enacted a number of COVID-relief legislation packages, including family-friendly policies such as the Families First Coronavirus Response Act (FFCRA) entitling a subset of employers with fewer than 500 employees to receive tax credits to partially offset the costs of providing leave benefits [16]. Some larger employers turned to existing policies such as the Family Medical Leave Act [FMLA] to provide employees with paid leave to assist with increased caregiving responsibilities; notably, only one-quarter of larger employers offer some sort of paid leave benefit even in non-pandemic times [17].

American employees more likely to have access to paid family leave benefits are full-time workers at large employers, higher-wage earners, and in management and professional occupations [18]. These employee characteristics disproportionally apply to more white men in higher income households than to women of any race or ethnicity, contributing to the inequities in the provision of critically needed paid leave benefits for mothers, especially mothers of color and lower income households [19]. One-third of the highest quartile of wage earners in the private sector has access to paid leave benefits compared to 8% of the lowest quartile of wage earners, providing evidence of the paradox that groups such as low income populations have one of the lowest rates of access to paid family leave benefits [18, 20].

Beyond the availability of paid leave for a minority of working mothers, other parental or family-friendly employment benefits have been enacted in a patchwork fashion across employers in the U.S. with the intention to ease strains around childcare and school closures, including flexible work location, flexible hours, and childcare assistance. For example, many professional-level workers have been given options to telecommute. This alternative potentially creates the unrealistic expectation that parents, especially mothers who disproportionately handle most of a household’s domestic workload, are able to complete both work responsibilities and meet the needs of children in virtual schooling environments [21]. Frontline workers such as most healthcare workers and low-waged, service industry workers do not have the option to telecommute, creating untenable scenarios of simultaneous work and childcare demands, especially among dual working parent families or single parent homes. Many individuals within these groups carry additional intersections as low-income households and as people of color [22]. This highlights one of the many inequalities exposed and exacerbated by the pandemic [23], along with discussions that rectifying these types of inequities and gaps in social safety nets should be continued as Americans emerge from the pandemic.

The provision of family-friendly or parental employment benefits such as paid family leave has been associated with improved mental health outcomes and reduced levels of chronic stress in pre-pandemic times [24, 25]. In 2004, California became the first state to enact paid family leave for working parents to care for a family member, providing 60 to 70% of wages for up to eight weeks within any 12-month period [26]. A longitudinal study measured mental health effects on parents prior to and after policy enactment, finding a sizable decrease in the levels of psychological distress for mothers at a time when they were eligible for paid family leave [27]. Other studies in the U.S. and abroad support the views that paid family leave is positively correlated with maternal mental health outcomes [25, 28, 29].

Additional benefits provided prior to the pandemic such as flexible work location and hours arrangements have also demonstrated positive maternal mental health outcomes such as reduced stress levels, lower rates of depression, and a greater sense of well-being, with much of the positive mental outcomes predicated by high levels of support and communication from managers and peers [30, 31]. Drawing evidence from Canada’s implementation of the universal childcare benefit (UCCB), childcare financial assistance and associated positive mental health outcomes serves as another example of the positive impacts of family-friendly employment benefits [32]. Canada’s UCCB, implemented in 2006, introduced a $100 CAD monthly payment per child under the age of six [32]. Mental health outcomes in a study leveraging Canadian population-level data showed the UCCB was associated with positive effects on maternal mental health, with stronger effects observed in single mothers [32]. Mental health impacts of similar child-related monetary benefits are evident in the expansion of the U.S. earned income tax credit benefit structure throughout the 1990s and 2000s and associations with maternal mental health [33]. Receipt of child-related earned income tax credits were associated with improved maternal mental health outcomes in both dual and single parent households, demonstrating that these types of safety nets not only positively influence financial instability issues, but concurrent mental health impacts [33].

While currently no studies have examined the longer-term mental health impacts of a scenario of the magnitude of the COVID-19 pandemic and related economic implications, experiences of populations impacted by previous pandemics and economic crises may be informative, including the 2003 outbreak of Severe Acute Respiratory Syndrome (SARS) and the Great Recession occurring between 2007 and 2009. SARS pandemic survivors showed elevated levels of stress during the outbreak that continued one year later without any indication of decreases; survivors showed higher levels of depression, anxiety, and posttraumatic symptoms than control subjects [34]. Individuals who experienced a negative job impact during the Great Recession demonstrated higher odds of poor mental health three to four years after the recession had ended [35]. Mothers in particular showed poor mental health outcomes in association with negative financial impacts during the recession [36, 37].

Although studies have assessed the mental health impact of the pandemic on U.S. adults, less is known about specific employment solutions that will support the mental health of working mothers. Additionally, working parents’ perceptions of and satisfaction with family-friendly employment benefits during the pandemic has not been widely studied. Given the challenges and burdens facing working mothers, additional research is needed to better understand working mothers’ mental health status approximately one year into the COVID-19 pandemic and potential associations with the receipt of family-friendly employment benefits that have positively influenced maternal mental health in non-pandemic times. The goal of the study was to assess the prevalence and type of employment benefits received by working mothers during the pandemic, understand levels of satisfaction with benefits provided, and evaluate working mothers’ mental health status after enduring approximately one year of balancing work and family during unprecedented times. Study outcomes not only provide an objective view of mental health associations with family-friendly benefits, but an understanding of the satisfaction with benefits provided from the perspective of working mothers. Without a clear plan for many mothers to return to work as they knew it prior to 2020, insights must be provided leading to possible employer and government policy pathways for further investigation, and the subsequent development of potential social, workplace, and government support strategies to ease associated burdens for working mothers and their families.

## Methods

### Study aims, design, and setting

The aim of this study was to investigate working mothers’ mental health status and associations with the types and prevalence of family-friendly employment benefits provided during the first year of the pandemic. In addition, the study aimed to identify subpopulations with an increased risk of mental health issues and associated gaps in workplace policy. Our study addressed the following research objectives:

1. To assess the prevalence and type of family-friendly employment benefits received by working mothers in the U.S. and working mothers’ perceptions of and satisfaction with employment benefits during the COVID-19 pandemic.
2. To examine the current mental health status of working mothers in the U.S and associations with the provision of family-friendly employment benefits during the COVID-19 pandemic approximately one year after the declaration of public health emergency.

A cross-sectional quantitative research study was performed using an anonymous online survey to collect data from U.S. working mothers. The survey consisted of 26 questions including validated and newly developed items and was open for responses for a period of two weeks between April 20, 2021 and May 3, 2021. The survey was created using Qualtrics, leveraging Qualtrics’ anonymity capabilities to ensure responses remained anonymous [38].

### Sample & recruitment

Through virtual social media platform ads, working mothers were invited to participate in the survey if they met the following inclusion criteria: (1) 18 years of age or older, (2) reside in the U.S., (3) have at least one child under the age of 18 in their household who they care for, and (4) have engaged in paid part- or full-time work for at least three months since the beginning of the COVID-19 pandemic. The online survey was distributed through social media platforms through paid ads, organic (unpaid) ads posted in working mother and moms’ groups, and by email outreach efforts to relevant individuals and organizations (e.g., public health professionals, maternal health and community organizations) for a period of two weeks. Virtual snowball sampling through sharing of paid Facebook ads, Facebook posts, and email outreach efforts was used to recruit additional survey participants. Sampling and recruitment through convenience and virtual snowball techniques leveraging Facebook is a cost-effective approach to reach populations such as working mothers in the U.S. [39]. As an incentive for participation, survey participants had the option at the conclusion of the survey to complete a separate, unlinked survey to capture contact information to be entered in a drawing for one of ten gift baskets valued at approximately 50 USD each.

In anticipation of a potential overrepresentation of young white females in survey responses, as has been demonstrated in previous health research studies leveraging Facebook for recruiting purposes, sociodemographic characteristics were monitored while the survey was open [40]. Survey response rates were reviewed to identify over- and under-representation of specific sociodemographic groups and to inform audience outreach approaches to ensure the inclusion of frequently underrepresented groups (e.g., people of color and lower income mothers). The most current data available on the U.S. workforce by race/ethnicity and the U.S. household income distribution ratio from the United States Bureau of Labor Statistics (BLS) and Statista, respectively, were used as baseline comparison ratios informing target audience adjustments [41, 42].

#### Sample size and response rates

The estimated sample size required to detect a two-point difference in the Kessler mental health scores was 120 participants based on similar populations mean scores, which would generate findings with a 95% confidence level, a 5% margin of error and a power of 80%. Given the social media outreach strategy, a traditional survey response rate was not able to be calculated. Of those participants who completed the screener, 96.2% (*N* = 866) were eligible to participate in the survey and 80.9% (*N* = 728) successfully completed the survey.

#### Ethical approval

All study methods were performed in accordance with the relevant guidelines and regulations under the George Washington University Committee of Human Research for ethical human subjects research. The study was approved and determined to be exempt by the George Washington University Committee of Human Research (Reference number: IRB NCR213382). An informed consent was displayed at the initiation of the survey to inform the participants of the purpose of the study, how the data will be used, and their rights as participants in the research project. Participants were assured that all information collected would remain confidential and used strictly for research purposes. Participants who provided consent and agreed to continue were asked screening questions at the beginning of the survey to determine if participants were eligible and could proceed with the data collection component of the questionnaire.

Due to the potentially sensitive nature of survey questions related to a participant’s mental health status participants had the option to discontinue the survey at any point by clicking a button indicating, “I would like to stop taking the survey” and have mental health and parenting assistance resources display such as the Crisis Text Line, National Suicide Prevention Lifeline and Circle of Parents support resources. Participants who completed the survey were also provided with the same mental health and parenting resources at the end of the survey.

### Description of measures

The online survey obtained responses from participants on self-reported measures of pandemic-related family-friendly employment benefits received, mental health status, sociodemographics, family characteristics, and work characteristics.

#### Independent variable: family-friendly employment benefit receipt

Pandemic-related, family-friendly employment benefits were measured by self-reported benefit receipt (yes/no) and by participants providing the benefit types received. Benefits included temporary increased wages, supplemental paid and unpaid time off, flexible hours/schedule to help with caregiving responsibilities, additional sick leave, flexible work location arrangements, flexible work location set-up, financial assistance, childcare financial assistance, mental health and well-being programs, and enhanced health insurance programs. A freeform field allowed participants to self-report benefit types not prepopulated in the survey. All freeform benefits provided were reviewed and allocated to suitable benefit categories for data analysis.

#### Independent variable: perceived helpfulness of benefits

Participant provided responses were used to determine a mean helpfulness score for each benefit on a Likert-scale of 0 (not at all helpful) to 4 (extremely helpful). All mothers, regardless of benefit receipt or non-receipt, were prompted to provide the Likert-scale benefit helpfulness score based on how helpful a benefit has been or could be based on their current employment experience. Collection of the benefit helpfulness scores from those who did and those who did not receive benefits informed the calculation of a subjective benefit helpfulness measure applicable to the full sample of diverse mothers. The benefit means scores allowed further investigation into which benefits had potentially stronger associations with mental health outcomes.

#### Dependent variables: maternal mental health

Mental health is a multidimensional state with constructs beyond psychological distress to consider when evaluating an individual's or population’s mental health status, so the study included assessments of both mental distress and a mental well-being. Psychological distress was obtained by the Kessler 6 (K-6) short-form screening scale to provide levels of psychological distress over the last 30 days [43]. The K-6 is widely regarded as a concise screening scale with good precision in identifying mental health disorders with a sensitivity of 0.34 and specificity of 0.96 at cut points of 5+ and 13+ to identify moderate and severe psychological distress, associated with moderate and serious mental illness, respectively [44]. The screening scale is used widely in the U.S. and abroad in surveys to measure mental health at the individual and community levels. In the self-administered version of the K-6 used in this study, participants self-reported a Likert-scale from 0 to 4 to six questions providing insights into their levels of psychological distress. Items were summed for a total score ranging from 0 to 24 and cut points of 5 and 13 were leveraged for assessing the prevalence of moderate mental illness (MMI) and serious mental illness (SMI), respectively [45]. Numeric scores were also retained as a continuous variable to provide more detailed findings regarding mental health status. Cronbach’s alpha demonstrated our use of the K-6 scale was highly reliable (α = 0.86).

To assess mental well-being, questions from the short version of the Warwick-Edinburgh Mental Wellbeing Scale (SWEMWBS) were included. The WEMWBS was developed to enable the examination of mental wellbeing in the general population through positively worded statements and Likert-scale responses [46]. Participants responded to seven items with responses ranging from 1 to 5 and a total raw score was calculated. Raw scores were transformed to metric scores as indicated by the Warwick Medical School’s scale use standards [47]. Scores from the SWEMWBS can be divided into high, average, and low mental wellbeing categories using the cut points of 28–35 and 7–19 for high and low mental wellbeing, respectively [47]. Continuous numeric scores were retained similarly to the K-6 scores for further analysis. Cronbach’s alpha demonstrated our use of the SWEMWBS scale was highly reliable (α = 0.84).

#### Covariates: Sociodemographics, family and work characteristics

Sociodemographics included age (18–34 years old, 35–44 years old, > 44 years old), race/ethnicity (Asian; Black, Indigenous, and People of Color (BIPOC); Hispanic/Latina; White), annual household income (less than $34,999, $35,000 to $49,999, $50,000 to $74,999, $75,000 to $99,999, $100,000 to $149,999, Over $150,000), highest level of education completed (some college or less, associate degree, bachelor’s degree, master’s degree, doctorate), and U.S. geographic region (Midwest, Northeast, South, West). BIPOC individuals were defined as those participants that self-reported Black, Indigenous American, or Alaskan Native as their race in the survey and were combined due to smaller sample sizes in these groups preventing the ability to report on each separately. Family characteristics included marital status (married or in a domestic partnership, or single, separated, divorced, or widowed), number of children under the age of 18 living in the household (one, two, three, four or more), the presence of children under the age of six living in the household (yes/no), level of disruption in childcare/schooling (never, rarely, sometimes, often, always). For data analysis, a significant disruption in childcare/schooling was categorized by responses of either often or always. Work characteristics included part-time or full-time work and work environment (home or flexible location, office or warehouse setting, educational establishment, health care establishment, setting with regular public interaction, other).

### Data analysis

For the first research objective, family-friendly employment benefit type prevalence measures were used to assess the receipt and distribution of benefits across the sample of working mothers. Descriptive statistics were examined for participants receiving and not receiving benefits across sociodemographic, family, and work covariates; chi-square tests were used for categorical covariates and two sample t-tests for continuous covariates. Participants’ perceptions of and satisfaction with employment benefits were measured by mean Likert-scale scores for each employment benefit type. Means for numbers of benefits received by categorical measures were tested using Analysis of Variance (ANOVA).

For the second research objective, descriptive statistics were compared between participants that did and did not report poor mental health using chi-square tests for categorical covariates and two sample t-tests for continuous covariates. Means for mental health measures were tested using Analysis of Variance (ANOVA). Frequencies of benefit types and means for reported benefit helpfulness also informed associations between mental health outcomes and benefits received. Finally, unadjusted and adjusted multivariable logistic regression was used to determine associations between psychological distress (K-6) or well-being (SWEMWBS) and family-friendly employment benefits receipt or non-receipt. Adjusted models included age, race/ethnicity, household income, educational attainment, and marital status. *P*-values less than 0.05 were considered significant. All analyses were conducted using SPSS software version 27.

## Results

### Characteristics of study participants by receipt of benefits

Of the 900 participants who responded to the survey, 172 participants did not meet study inclusion criteria or complete the questionnaire. In the final sample of 728 working mothers, 55.6% (*n* = 405) reported receiving one or more pandemic-related employment benefit during the last year. Participants who received benefit(s) were more likely to be between the ages of 34 and 44, report higher levels of education and higher levels of household income, reside in the West region of the U.S., currently work at home or at a flexible location, and be married or in a domestic partnership (Appendix: Table 2). Differences in receipt of benefits were not observed by race/ethnicity or part-time or full-time work status.

The number of benefits received varied by sociodemographics and work characteristics. The youngest mothers aged 18–34 years received the fewest benefits with a mean of 1.3 (*SD* ± 1.7) benefits per participant, while participants aged 34–44 years and those older than 44 years received an average of 1.7 (*SD* ± 1.8) and 1.5 (*SD* ± 1.8; *p* = 0.014) benefits, respectively. Overall increases in the number of benefits received were observed with increases in educational attainment and household income. Those completing some college or less received a mean of 0.6 (*SD* ± 1.1) benefits, while those holding bachelors, masters, or doctoral degrees received means of 1.7 benefits (*SD*s ± 1.7, 1.8, 1.8; *p* = 0.001). Participants with annual household incomes less than $34,000 received a mean of 0.6 (*SD* ± 1.1) benefits, while those at the top of the income scale making over $100,000 receiving a mean of 1.9 benefits (*SD* ± 1.8; *p* < 0.001). The number of benefits by work type and work environment was also statistically significant, with those working in education and healthcare receiving fewer benefits (*M* = 1.3, *SD* ± 1.6 and *M* = 1.0, *SD* ± 1.5) compared to those working in government or non-profit jobs (*M* = 2.1, *SD* ± 1.8 and *M* = 2.1, *SD* ± 1.7).

### Family-friendly benefit type receipt and perceived helpfulness

The family-friendly benefit most frequently received was flexible hours/schedule to help with caregiving, with 46.3% (*n* = 337) of participants reporting receipt (Table 1). Flexible work location and mental health and well-being programs were received by 37.4% (*n* = 272) and 22.7% (*n* = 165) of participants. Mean benefit perceived helpfulness scores were calculated for the total sample, those who received benefits, and those who did not receive benefits. All three mean helpfulness scores demonstrated that the three benefits perceived to be the most helpful for participants were flexible hours/schedule, flexible work location, and extra paid time off. The three benefits’ mean scores fell above 3.0 indicating that the benefits were perceived to be very helpful across the sample.

**Table 1:** Frequencies and means for employment benefit receipt and reported helpfulness

			Reported helpfulness score					
	Frequency of benefit receipt (N = 728)		Total sample (N = 728)		Did not receive benefit(s) ( n= 323)		Received ≥ 1 benefit(s) (n = 405)	
Family-friendly benefits	n	%	Mean	SD	Mean	SD	Mean	SD
Flexible hours/schedule to help with caregiving	337	(46.3)	3.5	0.9	3.3	1.0	3.6	0.7
Flexible work location	272	(37.4)	3.3	1.1	3.0	1.3	3.6	0.9
Extra paid time off	105	(14.4)	3.1	1.1	3.3	1.1	3.1	1.1
Childcare financial assistance	32	(4.4)	2.9	1.3	3.0	1.4	2.8	1.3
Temporary increased wages	20	(2.7)	2.9	1.2	2.9	1.2	2.6	1.3
Flexible work location set-up assistance	83	(11.4)	2.6	1.4	2.4	1.4	2.8	1.2
Extra sick days	97	(13.3)	2.6	1.7	2.7	1.3	2.4	1.3
Enhanced health insurance	15	(2.1)	2.5	1.4	2.7	1.4	2.4	1.4
Mental health well-being programs	165	(22.7)	2.3	1.3	2.4	1.3	2.2	1.3
Extra unpaid time off	36	(4.9)	1.5	1.7	1.4	1.5	1.4	1.3

### Mental health outcomes and associations with employment benefit receipt

The mean psychological distress (K-6) score for the full sample was 8.5 (*SD* ± 4.9), indicating the sample mean score fell above the cut point of 5+ for moderate psychological distress associated with MMI. Mothers who did not receive benefits had higher mean psychological distress scores (*M* = 9.2, *SD* ± 4.9) compared to mothers who received benefits (*M* = 7.9, *SD* ± 4.8, *p* < 0.001). As the number of benefits received increased among participants, the mean K-6 score decreased (*p* = 0.002). Statistically significant differences in K-6 mean scores were observed by mothers’ level of education attainment, household income, and disruption in childcare/schooling. Compared to their counterparts, higher mean K-6 scores were reported for mothers with lower levels of education attainment (*M* = 11.8, *SD* ± 5.0, *p* < 0.001), lower levels of household income (*M* = 10.6, *SD* ± 6.4, *p* = 0.001), and for those perceiving significant disruptions in childcare/schooling (*M* = 9.2, *SD* ± 4.9, *p* < 0.001).

The mean SWEMWBS score for the full sample was 20.2 (*SD* ± 3.3), placing the sample mean score in the average mental wellbeing category, slightly above the cut point of 19 for Low Mental Wellbeing (LMW). Statistically significant differences in mental well-being scores were found by race/ethnicity, benefit receipt, number of benefits received, and disruption in childcare/schooling. Reporting worse mental health on the SWEMWEBS than their counterparts were white working mothers (*M* = 20.0, *SD* ± 3.2, *p* = 0.033), participants who did not receive benefits (*M* = 19.8, *SD* ± 3.5, *p* = 0.013), and those who perceived a significant childcare/schooling disruption (*M* = 19.8, *SD* ± 2.6, *p* < 0.001).

Categorical mental health measures were evaluated using the widely accepted cutoff points for both the K-6 and SWEMWBS (Appendix – Table 3). The prevalence of any mental illness (i.e., MMI and SMI) for the sample was 76.1% (*n* = 554), with 21.8% (*n* = 159) meeting the psychological distress threshold for SMI. A higher prevalence of SMI was detected among participants reporting no benefits received compared to those receiving any benefit(s) (27.6% vs. 17.3%, *p =* 0.001). Four specific benefits were found to be associated with a significant lower prevalence of SMI among recipients compared to nonrecipients: flexible hours/schedule for caregiving (15.7% vs. 27.1%, *p <* 0.001), flexible work location (17.6% vs. 24.3%, *p =* 0.030), extra paid time off (15.2% vs. 23.0%, *p =* 0.045), and mental health and wellbeing programs (13.3% vs. 23.1%, *p =* 0.008). The prevalence of LMW in the sample was 39.0% (*n* = 284). A higher level of LMW was associated with participants not receiving benefits compared to those receiving benefits (44.0% vs. 35.1%, *p =* 0.050). Mothers with higher levels of mental wellbeing more often reported receipt of flexible hours/schedule (33.8% vs. 43.2%, *p =* 0.038) and mental health and wellbeing programs (30.9% vs. 39.3%, *p =* 0.049).

Separate multivariable logistic analyses assessed employment benefits and factors associated with SMI and LMW (Appendix – Table 4). In adjusted analyses, not receiving benefits was significantly associated with increased odds of SMI (*aOR* = 1.50, *CI* [1.03–2.20], *p* = 0.036) and LMW (*aOR* = 1.38, *CI* [1.00–1.89], *p* = 0.049). Mothers who received benefits showed decreased odds of SMI as the number of benefits received increased (*aOR* = 0.50, *CI* [0.27–0.93], *p* = 0.031). Not receiving two specific benefits were significant predictors of SMI: flexible hours/schedule (*aOR* = 1.64, *CI* [1.10–2.42], *p* = 0.014) and mental health and wellbeing programs (*aOR* = 1.72, *CI* [1.03–2.86], *p* = 0.037). Not receiving flexible hours/schedule was also associated with LMW (*aOR* = 1.40, *CI* [1.02–1.93], *p* = 0.038).

## Discussion

This study is one of the first to focus on working mothers and their mental health status, approximately one year after both the acute dangers of COVID-19 became clearly apparent and school closures were at their peak [48]. At the time of data collection in April and May 2021, the U.S. was experiencing a decline in infections, progress with vaccinations, a steady return to in-person schooling, and an unemployment rate for women of 5.1% compared to that of 13.6% at the same time one year earlier [12, 13, 45]. Despite progress in a reduction of infections, more reliable childcare/schooling options, and many women returning to employment, working mothers’ mental health is still suffering as described by our findings.

Several studies have examined mental health impacts of the COVID-19 pandemic on the U.S. population and subpopulations of women and mothers during various perceived peaks of the pandemic in 2020 and found that the mental health across the country declined [5, 49, 50]. The magnitude and prevalence of mental illness among working mothers presented in our research closely mirrors the levels of psychological distress in the U.S. population in April 2020, approximately one month after stay-at-home orders upended almost all routines for Americans. At that time, 71.4% of women fit the criteria for MMI or SMI, and 27.2% of women fit the criteria for SMI [50].

More recently, longitudinal studies of a nationally representative population found an initial rise in psychological distress among Americans in April and May of 2020 and a subsequent fall back to baseline later in 2020 [51, 52]. These studies postulate that a level of resilience within the population, along with swift governmental action to provide financial support for many Americans, potentially mitigated further mental health deterioration among much of the population. Another study, focusing on the mental health trajectories in UK adults during the pandemic arrived at similar findings, with most adults returning to pre-pandemic levels of mental health by October 2020 [53]. The findings of our study reveal the unlikelihood of a return to a mental health baseline for working mothers as high levels of psychological distress persist in this subpopulation.

In addition to examining the overall mental health status of U.S. working mothers, our study was one of the first to assess the provision of pandemic-related, family-friendly employment benefits during the COVID-19 pandemic and the associations with working mothers’ mental health outcomes. Not receiving family-friendly employment benefits was significantly associated with maternal SMI. Working mothers who received benefits showed reduced odds of SMI as the number of benefits received increased. Receiving more benefits was associated with higher education attainment and household income; this finding corresponds with BLS data as of March 2020, indicating that many workplace benefits were more often provided to the top versus bottom quartile of wage earners (medical benefits: 93% vs. 41%, paid leave: 94% vs. 52%, flexible workplace: 18% vs. 1%) [54]. The contrast between lower wage workers receiving few or no benefits and higher wage workers receiving higher numbers of benefits supports the need to mitigate this inequality highlighted by the pandemic.

Work schedule flexibility, location flexibility, and mental health and wellbeing programs demonstrated promise in driving better mental health in our adjusted models. Flexibility in work arrangements have been linked to better mental health outcomes among employees in pre-pandemic times and have become a recurrent discussion topic in the context of mental health as the U.S. begins its emergence from the pandemic, with employer support cited as a critical component necessary to reap mental health benefits [55, 56]. Mean helpfulness scores for mental health and wellbeing programs in the current study was marginally above the “somewhat helpful” threshold, yet associations with improved mental health outcomes were significant. The link between provision of mental health and wellbeing programs and better mental health outcomes warrants further investigation to understand the types and usage of programs yielding positive mental health benefits.

Paid time off was associated with a lower prevalence of SMI among participants and was reported as one of the most helpful benefits among working mothers. However, paid time off was no longer significantly associated with SMI in our adjusted models controlling for sociodemographic variables (i.e., age, race/ethnicity, household income, educational attainment, and marital status). Our mixed results suggest additional research is necessary to better understand the mental health benefits of paid leave across sociodemographic segments of working mothers. Existing literature has demonstrated physical and mental health benefits of paid family leave and should be considered in tandem with our mixed findings when determining family-friendly employment benefit policies to improve the work experience and related mental health impacts for mothers [57–59].

This study had several limitations. First, while snowball sampling through social media platforms is a rapid, cost-effective means of reaching target populations, there are limitations to this sampling approach. Participant response rates could not be calculated, and participants could have taken the survey more than once, as the survey was anonymous. A potential low response rate could impact the generalizability of the findings, and the participating sample may be biased. BIPOC and Latina mothers were underrepresented in the study sample; the survey was only available in English which may have had a negative impact in recruiting Latina Americans. Lower household income segments were underrepresented as well. Lower income individuals may not have the time or resources to dedicate to taking the survey, potentially resulting in self-selection bias. Furthermore, working mothers suffering from poor mental health may have been more likely to complete the survey, again resulting in self-selection bias. Household size was not collected preventing the ability to adjust household income by number of family members per household. The cross-sectional study design allows the simultaneous observation of exposures and outcomes, while longitudinal studies are necessary and recommended for future research to examine the temporal relationship between benefits and mental health outcomes. Finally, statistical models adjusted for potential confounders, yet some confounders may still present limitations in the ability to generalize findings across the full population of 21.7 million working mothers in the U.S. [4].

## Conclusion

The lack of statutory family-friendly employment benefits across populations of all backgrounds in the U.S. is just one of the many inequalities and vulnerabilities exposed and exacerbated by the pandemic [3, 23, 60]. Employment-related benefits and unemployment safety nets have demonstrated value with increases in met health-related social and economic needs and decreases in poor mental health [15, 25, 32]. Discussions and policy mitigations that rectify these types of inequalities and gaps in social safety nets should continue beyond the country’s emergence from the pandemic, as has been signaled by the current federal administration’s recent efforts. The American Jobs Plan, introduced on March 31, 2021 included a proposal for the allocation of $25 million to create more childcare facilities in the most high-need areas and to build on-site childcare facilities at places of work, creating flexible childcare options for those mothers needing it most [61].

Statutory paid family leave has been discussed on numerous occasions in the recent past, with President Biden addressing Congress in late April 2021 endorsing the American Families Plan legislation [62]. The American Families Plan initially proposed 12 weeks of paid family leave for parental, caregiving, and personal medical leave. The economic, physical, and mental health benefits of paid family leave would finally place the country in the same arena as all other developed nations regarding family-friendly benefits.

Mainstream media and grey literature have brought the mental health crisis for working mothers to attention through anecdotal stories accompanied with relevant statistics such as the number of women that have left the workforce as a result of the pandemic [63, 64]. However, empirical evidence examining the associations between mental health status of working mothers in the U.S. and provision of employment benefits was lacking. This study fills an important gap to better understand working mothers’ mental health status and associations with critical family-friendly employment benefits approximately one year after the declaration of the global public health emergency. Findings contribute to the body of evidence necessary for employer and government policy efforts to place working mothers in a position to rebound psychologically, socially, and economically in the post-pandemic environment. Findings also serve as evidence demonstrating the fragility of American working mothers’ circumstances and the urgent need for permanent, comprehensive, statutory family-friendly employment benefits, such as flexibility in work schedules and location, paid family leave, and mental health programs as necessities that have been called out in this time of global crisis.

## Data Availability

The datasets used and/or analyzed during the current study are available from the corresponding author on reasonable request.

## Appendix

**Table 2:** Sample characteristics by receipt of employment benefits

	Overall sample		Did not receive benefit(s)		Received ≥ 1 benefit(s)		Effect size^a^	P-value^b^
	n	%	n	%	n	%		
Total	728	100%	323	44.4%	405	55.6%	–	–
*Demographics*								
Age								
18–34 years old	185	25.4%	98	53.0%	87	47.0%	0.111	**0.012**
35–44 years old	445	61.1%	179	40.2%	266	59.8%		
> 44 years old	98	13.5%	46	46.9%	52	53.1%		
Race/ethnicity								
Asian	51	7.0%	25	49.0%	26	51.0%	0.028	0.906
Black, Indigenous, and People of Color (BIPOC)	44	6.0%	20	45.5%	24	54.5%		
Hispanic/Latina	24	3.3%	11	45.8%	13	54.2%		
White	609	83.7%	267	43.8%	342	56.2%		
**Highest level of education**								
Some college or less	40	5.5%	31	77.5%	9	22.5%	0.172	**< 0.001**
Associate degree	28	3.8%	15	53.6%	13	46.4%		
Bachelor’s degree	182	25.0%	70	38.5%	112	61.5%		
Master’s degree	334	45.9%	143	42.8%	191	57.2%		
Doctorate	144	19.8%	64	44.4%	80	55.6%		
**Annual household income**								
Less than $34,999	38	5.2%	29	76.3%	9	23.7%	0.262	**< 0.001**
$35,000 to $49,999	25	3.4%	20	80.0%	5	20.0%		
$50,000 to $74,999	80	11.0%	44	55.0%	36	45.0%		
$75,000 to $99,999	80	11.0%	43	53.8%	37	46.3%		
$100,000 to $149,999	169	23.2%	62	36.7%	107	63.3%		
Over $150,000	336	46.2%	125	37.2%	211	62.8%		
**Geographic Region**								
Midwest	134	18.4%	75	56.0%	59	44.0%	0.121	**0.013**
Northeast	318	43.7%	128	40.3%	190	59.7%		
South	186	25.5%	85	45.7%	101	54.3%		
West	90	12.4%	35	38.9%	55	61.1%		
***Work Characteristics***								
**Work Hours**								
Full-time	621	85.3%	270	43.5%	351	56.5%	0.043	0.244
Part-time	107	14.7%	53	49.5%	54	50.5%		
**Work environment**								
Home or flexible location	339	46.6%	126	37.2%	213	62.8%	0.219	**< 0.001**
Office or warehouse setting	179	24.6%	70	39.1%	109	60.9%		
Educational establishment	56	7.7%	30	53.6%	26	46.4%		
Health care establishment	100	13.7%	67	67.0%	33	33.0%		
Setting with regular public interaction	36	4.9%	21	58.3%	15	41.7%		
Other	18	2.5%	9	50.0%	9	50.0%		
**Marital Status**								
Married or in a domestic partnership	656	90.1%	281	42.8%	375	57.2%	0.093	**0.012**
Single, separated, divorced, or widowed	72	9.9%	42	58.3%	30	41.7%		

**Table 3:** Frequencies of Kessler-6 and SWEMWBS mental health categories

		Kessler 6								SWEMWBS							
		No Mental Illness		MMI		SMI				High Mental Wellbeing		Average Mental Wellbeing		Low Mental Wellbeing			
Characteristic	Total	n	%	n	%	n	%	Effect size^a^	P-value^b^	n	%	n	%	n	%	Effect size^a^	P-value^b^
***Benefits Characteristics***																	
**Employer provided benefits**																	
Yes	323	63	(19.5)	171	(52.9)	89	(27.6)	0.136	**0.001**	7	(2.2)	174	(53.9)	142	(44.0)	0.091	**0.050**
No	405	111	(27.4)	224	(55.3)	70	(17.3)			11	(2.7)	252	(62.2)	142	(35.1)		
**Number of Benefits Received**																	
None	323	63	(19.5)	171	(52.9)	89	(27.6)	0.119	**0.009**	7	(2.2)	174	(53.9)	142	(44.0)	0.087	0.196
One	61	11	(18.0)	36	(59.0)	14	(23.0)			0	(0.0)	35	(57.4)	26	(42.6)		
Two	111	28	(25.2)	64	(57.7)	19	(17.1)			3	(2.7)	67	(60.4)	41	(36.9)		
Three	118	38	(32.2)	58	(49.2)	22	(18.6)			3	(2.5)	77	(65.3)	38	(32.2)		
Four or more	115	34	(29.6)	66	(57.4)	15	(13.0)			5	(4.3)	73	(63.5)	37	(32.2)		
**Receipt of Benefit Type**																	
Flexible hours/schedule to help with caregiving																	
Yes	337	96	(28.5)	188	(55.8)	53	(15.7)	0.151	**<0.001**	10	(3.0)	212	(62.9)	114	(33.8)		0.038
No	391	78	(19.9)	207	(52.9)	106	(27.1)			8	(2.0)	214	(54.7)	169	(43.2)		
Flexible work location																	
Yes	272	77	(28.3)	147	(54.0)	48	(17.6)	0.098	**0.030**	9	(3.3)	166	(61.0)	97	(35.7)	0.064	0.229
No	456	97	(21.3)	248	(54.4)	111	(24.3)			9	(2.0)	260	(57.0)	187	(41.0)		
Extra paid time off																	
Yes	105	34	(32.4)	55	(52.4)	16	(15.2)	0.092	**0.045**	4	(3.8)	68	(64.8)	33	(31.4)	0.069	0.173
No	623	140	(22.5)	340	(54.6)	143	(23.0)			14	(2.2)	358	(57.5)	251	(40.3)		
Mental health well-being programs																	
Yes	165	47	(28.5)	96	(58.2)	22	(13.3)	0.115	**0.008**	4	(2.4)	110	(66.7)	51	(30.9)	0.091	**0.049**
No	593	127	(21.4)	299	(50.4)	137	(23.1)			14	(2.4)	316	(53.3)	233	(39.3)		
**Work hours**																	
Full-time	621	145	(23.3)	346	(55.7)	130	(20.9)	0.072	0.151	13	(2.1)	360	(58.0)	248	(39.9)	0.070	0.166
Part-time	107	29	(27.1)	49	(45.8)	29	(27.1)			5	(4.7)	66	(61.7)	36	(33.6)		

Kessler 6 cut points for MMI (Moderate Mental Illness), and SMI (Severe Mental Illness) were 5+ and 13+, respectively; higher scores indicate higher levels of psychological distress; SWEMWBS cut points for High and Low Mental Wellbeing were of 28–35 and 7–19, respectively; lower scores indicate higher levels of mental wellbeing

^a^ Effect size calculated using Cramer’s V

^b^ P-value based on Chi-squared tests; Statistically significant p-values bolded

**Table 4:** Unadjusted and adjusted odds ratios of Serious Mental Illness and Low Mental Wellbeing

	Kessler 6					SWEMWBS				
	Serious Mental Illness					Low Mental Wellbeing				
Characteristic	OR	95% CI	AOR	95% CI	p-value	OR	95% CI	AOR	95% CI	p-value
**Employer provided benefits**										
Yes	Reference									
No	1.82	(1.28–2.60)	1.50	(1.03–2.20)	**0.036**	1.45	(2.22–2.22)	1.38	(1.00–1.89)	**0.049**
**Number of benefits received**										
None	Reference									
One	0.78	(0.41–1.49)	0.91	(0.46–1.74)	0.775	0.95	(0.55–1.65)	0.98	(0.56–1.74)	0.958
Two	0.54	(0.31–0.94)	0.64	(0.35–1.14)	0.124	0.75	(0.48–1.16)	0.77	(0.49–1.21)	0.262
Three	0.60	(0.36–1.02)	0.72	(0.41–1.14)	0.231	0.61	(0.39–0.94)	0.65	(0.41–1.03)	0.065
Four or more	0.39	(0.22–0.72)	0.50	(0.27–0.93)	**0.031**	0.60	(0.39–0.95)	0.64	(0.39–1.02)	0.062
**Employer provided benefit received**										
*Flexible hours/schedule to help with caregiving*										
Yes	Reference									
No	1.99	(1.38–2.88)	1.64	(1.10–2.42)	**0.014**	1.47	(1.09–2.29)	1.40	(1.02–1.93)	**0.038**
*Flexible work location*										
Yes	Reference									
No	1.47	(1.09–1.99)	1.25	(0.83–1.86)	0.283	1.25	(0.92–1.71)	1.17	(0.85–1.63)	0.336
*Extra paid time off*										
Yes	Reference									
No	1.66	(0.94–2.91)	1.55	(0.87–2.77)	0.137	1.47	(0.95–2.29)	1.49	(0.95–2.34)	0.084
*Mental health and wellbeing programs*										
Yes	Reference									
No	2.09	(1.28–3.41)	1.72	(1.03–2.86)	**0.037**	1.58	(1.09–2.29)	1.45	(0.99–2.14)	0.059
**Work environment**										
Home or flexible location	Reference									
Office or warehouse setting	0.85	(0.54–1.35)	0.80	(0.50–1.27)	0.345	0.98	(0.68–1.42)	0.95	(0.65–1.38)	0.781
Educational establishment	1.33	(0.70–2.54)	1.05	(0.23–2.09)	0.888	1.15	(0.65–2.03)	0.98	(0.54–1.78)	0.946
Health care establishment	0.59	(0.32–1.10)	0.57	(0.30–1.08)	0.083	0.75	(0.47–1.21)	0.72	(0.44–1.18)	0.190
Setting with regular public interaction	3.26	(1.61–6.60)	2.17	(1.00–4.69)	**0.049**	1.22	(0.61–2.25)	0.98	(0.47–2.07)	0.966
Other	1.82	(0.66–5.02)	1.04	(0.34–3.17)	0.940	0.97	(0.37–2.57)	0.50	(0.25–1.97)	0.702
**Age**										
18–34 years old	Reference									
35–44 years old	1.25	(0.81–1.94)	1.50	(0.95–2.38)	0.080	1.08	(0.76–1.54)	1.18	(0.82–1.70)	0.372
> 44 years old	1.69	(0.95–3.01)	1.76	(0.94–3.29)	0.076	0.85	(0.51–1.42)	0.91	(0.53–1.56)	0.732
**Race / Ethnicity**										
White	Reference									
Asian	1.25	(0.65–2.41)	1.56	(0.78–3.11)	0.205	0.67	(0.36–1.24)	0.76	(0.41–1.43)	0.395
Black, Indigenous, and People of Color (BIPOC)	0.94	(0.44–2.00)	0.70	(0.31–1.58)	0.389	0.49	(0.24–0.99)	0.44	(0.21–0.91)	**0.027**
Hispanic/Latina	1.22	(0.47–3.13)	1.00	(0.37–2.72)	1.000	1.05	(0.46–2.39)	0.94	(0.40–2.21	0.892
**Annual household income**										
Less than $34,999	Reference									
$35,000 to $49,999	0.87	(0.32–2.41)	0.95	(0.33–2.75)	0.930	1.57	(0.57–4.34)	1.42	(0.49–4.08)	0.511
$50,000 to $74,999	0.37	(0.16–0.84)	0.41	(0.16–1.02)	0.056	0.91	(0.42–1.98)	0.83	(0.35–2.00)	0.680
$75,000 to $99,999	0.42	(0.19–0.94)	0.44	(0.17–1.17)	0.100	1.12	(0.52–2.43)	0.99	(0.39–2.49)	0.989
$100,000 to $149,999	0.25	(0.12–0.53)	0.27	(0.11–0.69)	**0.007**	0.77	(0.38–1.57)	0.71	(0.29–1.73)	0.452
Over $150,000	0.23	(0.11–0.46)	0.22	(0.09–0.57)	**0.002**	0.65	(0.33–1.28)	0.61	(0.25–1.48	0.276
**Significant disruption in access to childcare/schooling**										
Yes	Reference									
No	1.74	(1.19–2.54)	1.96	(1.31–2.95)	**0.001**	1.63	(1.20–2.23)	1.64	(1.18–2.26)	**0.003**

Statistically significant p-values bolded

Serious Mental Illness was defined with the following widely accepted criteria: Kessler 6 psychological distress score ≥ 13; Low Mental Wellbeing was defined with the following widely accepted criteria: SWEMWBS score of 7–19 Adjusted logistic regression models included the following independent variables: age, race/ethnicity, household income, educational attainment, and marital status

## Abbreviations

ANOVA: Analysis of Variance
BIPOC: Black, Indigenous, and People of Color
BLS: United States Bureau of Labor Statistics
FFCRA: Families First Coronavirus Response Act
K-6: Kessler 6 Psychological Distress Scale
LMW: Low Mental Wellbeing
MMI: Moderate Mental Illness
SARS: Severe Acute Respiratory Syndrome
SMI: Serious Mental Illness
SWEMWBS: Short Warwick-Edinburgh Mental Wellbeing Scale
UCCB: Universal Child Care Benefit
